# Ketogenic diet and behavior: insights from experimental studies

**DOI:** 10.3389/fnut.2024.1322509

**Published:** 2024-02-08

**Authors:** Konstancja Grabowska, Mateusz Grabowski, Marta Przybyła, Natalia Pondel, Jarosław J. Barski, Marta Nowacka-Chmielewska, Daniela Liśkiewicz

**Affiliations:** ^1^Laboratory of Molecular Biology, Institute of Physiotherapy and Health Sciences, Academy of Physical Education, Katowice, Poland; ^2^Department for Experimental Medicine, Faculty of Medical Sciences in Katowice, Medical University of Silesia, Katowice, Poland; ^3^Department of Physiology, Faculty of Medical Sciences in Katowice, Medical University of Silesia, Katowice, Poland; ^4^Institute of Diabetes and Obesity, Helmholtz Center Munich, Neuherberg, Germany; ^5^German Center for Diabetes Research (DZD), Neuherberg, Germany

**Keywords:** ketogenic diet, cognition, depressive-like behavior, anxiety-like behavior, social behavior, nutritional behavior, nutritional ketosis, animal models

## Abstract

As a journal page for full details. The ketogenic diet (KD) has been established as a treatment for epilepsy, but more recently it has been explored as an alternative or add-on therapy for many other diseases ranging from weight loss to neurological disorders. Animal models are widely used in studies investigating the therapeutic effects of the KD as well as underlying mechanisms. Especially in the context of neurological, psychiatric, and neurodevelopmental disorders essential endpoints are assessed by behavioral and motor tests. Here we summarized research evaluating the influence of the KD on cognition, depressive and anxiety-related behaviors, and social and nutritional behaviors of laboratory rodents. Each section contains a brief description of commonly used behavioral tests highlighting their limitations. Ninety original research articles, written in English, performed on mice or rats, providing measurement of blood beta-hydroxybutyrate (BHB) levels and behavioral evaluation were selected for the review. The majority of research performed in various disease models shows that the KD positively impacts cognition. Almost an equal number of studies report a reduction or no effect of the KD on depressive-related behaviors. For anxiety-related behaviors, the majority of studies show no effect. Despite the increasing use of the KD in weight loss and its appetite-reducing properties the behavioral evaluation of appetite regulation has not been addressed in preclinical studies. This review provides an overview of the behavioral effects of nutritional ketosis addressed to a broad audience of scientists interested in the KD field but not necessarily specializing in behavioral tests.

## 1 Introduction

The ketogenic diet (KD) is a very low-carbohydrate, high-fat, and adequate protein nutritional approach that induces a metabolic shift to the use of ketone bodies as an additional energy source (1, 2). In the 1920s, physicians introduced the KD as a treatment for epilepsy, especially in patients poorly responding to pharmacotherapy. By the end of the XX century, the KD resurfaced, gaining popularity with the general public mainly due to its efficiency in treating obesity (3). Scientific interest in the KD also significantly increased, as illustrated by the fact that a PubMed search for ‘ketogenic diet’ shows 254 results until 2000 and 4,682 hits in the years 2000–2023. As a result of extensive research, it is now well established that, besides the well-known metabolic effects including ketosis and decreased blood glucose levels, the KD influences inflammatory processes, oxidative stress, gut microbiota, and intracellular signaling pathways (4–6). The KD also has a pleiotropic impact on brain functioning, including gene expression (7–9), neurotransmission (10), the level of neurotrophic factors (11, 12), protein phosphorylation (13), and the metabolism of amino acids (14). Due to this multifaceted effect on physiology, the KD has been increasingly investigated as an alternative or add-on therapy for many diseases (15, 16). Findings from large-scale clinical trials remain limited, and animal models are widely utilized in studies investigating the therapeutic effects of the KD as well as underlying mechanisms. Especially in the context of neurological, psychiatric, and neurodevelopmental disorders, essential endpoints are evaluated by behavioral tests, including the assessment of cognitive functions, and behaviors related to anxiety and depression. The examination of social behavior is important in studies related to Autism Spectrum Disorders (ASD). The reduction of appetite is considered crucial for the effectiveness of the KD in treating obesity in humans (17, 18). Therefore, the influence of the ketogenic diet on nutritional behavior is another interesting aspect that can be explored using animal models.

While there are several important considerations when designing behavioral experiments in animal models, one particularly crucial aspect in the KD field is the composition of the chow. Numerous variations of ketogenic chows are employed in animal research, with the most significant distinctions revolving around the macronutrient ratio, source of fat as well as macronutrient and vitamin content (19, 20). Appropriately chosen macronutrient ratio, not only carbohydrate restriction but also adequate protein content, determines the level of ketosis (21). In addition, particular fat content like medium chain triglycerides (MCT) can enhance ketone production (22–24). Since possible nutrient deficiencies resulting from a very restrictive diet are a common adverse effect of KD in humans (25), currently, a lot of attention is given to the composition of a dietary plan. The same should apply to animal models, where proper micronutrient supplementation of the ketogenic chow is critical to avoid adverse effects such as weakness and growth inhibition (26, 27).

Here, we review studies examining the influence of KD on the behavior of laboratory rodents. Included in the review were only studies that reported the level of ketosis, ensuring a minimal requirement in terms of diet composition for achieving nutritional ketosis. The article is organized into sections dedicated to cognition, depressive and anxiety-related behaviors, as well as social and nutritional behavior. Each section begins with a concise explanation of the rationale for assessing specific behaviors within the context of nutritional ketosis. Evaluation of animal behavior, interpretation of results, and translation of the findings to the clinically relevant situation require an understanding of the behavioral tests employed and inherent their limitations. Consequently, each section offers a brief overview of the available methods for evaluating the behavior in question, highlighting both limitations and potential caveats in result interpretation.

## 2 Search strategy and study selection

The term “ketogenic diet” was searched in PubMed without a year-of-publication restriction identifying 4,918. Only articles written in English were included. Articles were divided among all authors for screening of titles and abstracts in order to select full-text original papers conducted on mice or rats. In this first step of screening 880 publications were included and full texts were independently reviewed by two investigators in order to select studies showing behavioral or functional tests investigating: depressive-, and anxiety-related behaviors, cognition, and social behavior. One hundred and forty-five studies were selected for evaluation of eligibility. Separate screening was performed to identify studies reporting food intake which was discussed as an approximate of nutritional behavior.

**Figure 1:**
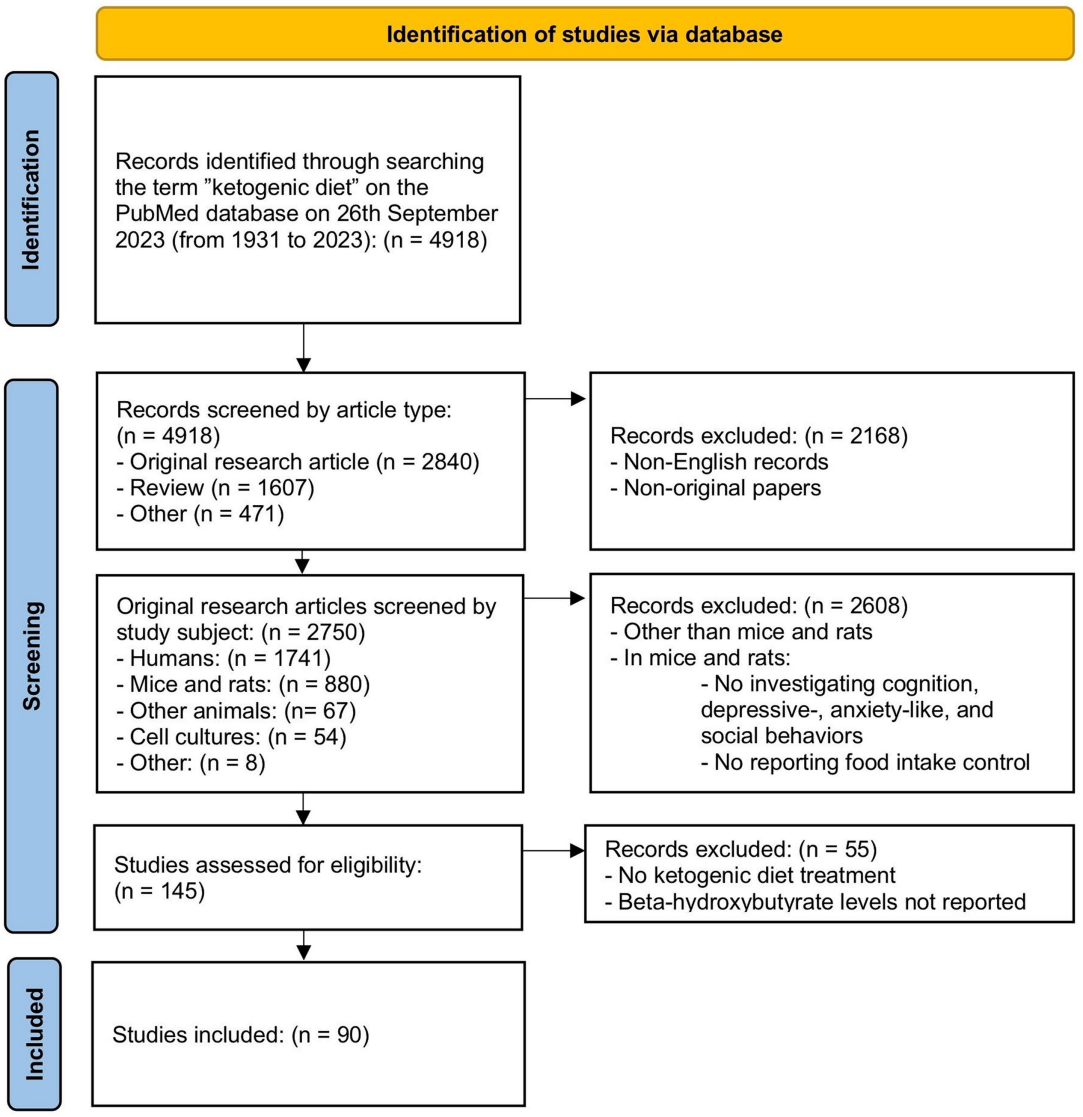
Flow chart showing the procedure of studies selection included in review.

This screening resulted in the identification of 65 articles. Only studies reporting the level of ketosis, as a measurement of blood beta-hydroxybutyrate (BHB) level, were included. Finally, 90 articles were included in the review. The last search was performed on September 26th, 2023. The flow chart illustrating the article selection process is shown in Figure 1.

## 3 Behavioral effects of KD treatment

### 3.1 Cognition

Nutritional ketosis reduces neuroinflammation, and oxidative stress, and improves mitochondrial function (28, 29). All these processes have a profound impact on brain health and in turn on neurocognitive functions. The neuroprotective properties of KDs were demonstrated in animal models of epilepsy, aging, dementia, and neurodegenerative diseases (30–32).

Cognition is a very complex construct that encompasses several aspects of intellectual functioning. Therefore, results arising from animal studies can only capture some of the meanings of this term.

Despite the availability of a variety of tests that measure slightly different, but also overlapping and interacting aspects of cognition, obtaining reliable and disease-relevant results requires careful choice of methods and usually using a combination of different paradigms.

The most commonly used behavioral test to measure impairment of cognitive functions is the Morris water maze (MWM). The MWM paradigm is designed to assess spatial learning and long-term memory by observing and recording escape latency, thigmotaxis duration, distance moved, and velocity during the time spent in the circular water tank with a hidden platform, where rodents are required to find the escape route to the platform by remembering visual cues (33). Another common test to evaluate learning and memory, particularly recognition memory, is the novel object recognition test (NOR). NOR test uses innate preference to interact with unknown objects in relation to known objects (e.g., blocks, balls) (34). Other behavioral tests used in animal studies involve modifications of maze tasks, for example, Y-maze, T-maze, V-maze, Hebb Williams Maze, or Barnes Maze. Above-described tests can be used to assess the rodents’ short- and long-term memory, learning, and spatial learning (35). It is important to note that the tests evaluating cognitive functions depend on the exploratory behavior of animals and their interaction with the environment. Other parameters like locomotor activity or levels of anxiety can significantly impact the outcomes of cognitive performance during tests, in addition to cognitive abilities themselves. In some paradigms, palatable food is used as a reward for conditional learning. Using these tests when comparing animals fed with KD or standard diet requires careful consideration since the animals fed with different diets may have various levels of motivation towards obtaining food. Moreover, an inappropriately selected snack may influence the level of ketosis.

The impact of KD on cognitive functions is most commonly described in epilepsy (37–44), traumatic brain injury (TBI) (45–49), Alzheimer’s disease (AD) (50–54), and also in healthy animals (55–62) especially in the context of aging (63–72).

In the animal models of epilepsy, it was reported in nine research articles (37–44). Despite the indisputable anti-seizure effects of KD, these studies do not bring unequivocal results regarding cognitive function. In different animal models of epilepsy: electrically elicited (kindled) seizures (36), spontaneously epileptic Kcna1-null mice (37), the pilocarpine-induced status epilepticus (38), and the pentylenetetrazol (PTZ)-kindled model (39) KD treatment led to improved memory functions. In the PTZ-kindled model of epilepsy KD improved spatial memory in the novel placement recognition test in rats, without changes in memory acquisition based on the MWM test results (39). Su et al. (40) drew attention to the importance of the timing of KD initiation in the pharmacological model of epilepsy, showing that early KD initiation (2 days after status epilepticus) resulted in weaker spatial learning in the MWM than observed in rats on a control diet or rats that started the KD 2 weeks after status epilepticus. In the context of epilepsy seizure activity is a primary cause of cognitive deficits (73, 74). Therefore, it is possible that the cognition-enhancing effect of the KD observed in the models of epilepsy is an indirect result of seizure mitigation. However, despite effective seizure mitigation, no improvements in cognitive performance after KD treatment were observed in other studies in a genetic model for idiopathic epilepsy (42), kindling model of epilepsy (43), and Dravet syndrome model (44). One study conducted in the lithium pilocarpine model of epilepsy showed substantially worsened performances in the MWM of young rats treated with KD. The severe impairment in visual–spatial memory was accompanied by decreased brain growth (41). Interestingly, when applying the ketogenic chow of the same composition to rats just after weaning, we observed adverse effects such as weakness, growth inhibition, and brain undergrowth. These adverse effects were mitigated when we supplemented the diet with wheat bran (27). Similarly, other authors achieved a significant reduction in adverse effects of the same ketogenic chow through supplementation with choline or methionine (26). It is important to mention that the same ketogenic chow (Bio-Serv F3666) used in mice, for example, in spontaneously epileptic Kcna1-null mice (37), in a TBI model (47) or in naive mice, led to improved cognitive measures. These studies are discussed in the further part of this section.

In the context of aging, healthspan, and lifespan numerous studies demonstrated improved cognitive function in KD-fed animals (63–72). KD treatment introduced at both old (20 months) and young (4 months) ages, enhanced cognition across the lifespan, regardless of sex. It resulted in enhanced performance on both the elevated figure-8 maze alternation task and a cognitive dual task that involved working memory. Also, the authors noted that the observed differences in protein expression related to metabolism and vesicular transport in the prefrontal cortex and hippocampus could contribute to the development of further therapies for age-related cognitive decline (68). Zhou et al. (63) showed that KD introduced at 18 months of age improved spatial learning and memory at 26 months of age. Roberts et al. (65) showed that aged male mice fed with KD show memory improvement in the NOR test as compared to the control but also to low-carbohydrate diet-fed animals. Authors suggested that nutritional ketosis, rather than the low glycemic index, affects index lifespan and slows down age-related cognitive impairment in old mice. In aged female mice after 2 months of KD, an improvement in spatial learning but no recognition memory or short-term working memory was observed (69). Newman et al. (64) reported that long-term exposure to the cyclic KD (given every other week) reduces mid-life mortality and preserves memory in aging males. Similarly, Hernandez et al. (67) found that time-restricted access to the KD in middle-aged mice positively affected cognitive functions compared to animals fed *ad libitum* with standard chow. Additionally, there were significant differences in gut microbiome diversity and composition in both diets. Authors suggested that improved cognition was associated with an altered gut microbiome, especially lowered *Allobaculum* abundance. Observed improvement may result from time-restricted feeding paradigm rather than macronutrient composition of KD and standard chow (67). These results provide evidence that the KD may beneficially affect cognitive function in female and male rodents, especially in middle-aged and old animals, not only after long-term exposure to diet (64, 65, 67–69), but also after a few weeks of treatment (66). On the other hand, exposure to KD from postnatal day (P) 20 - P32 for at least 5 weeks may adversely impact cognition later in life in laboratory rodents (40, 41, 71). Recently, Miles and Skelton (72) reported that early-life (from P21 through young adulthood ~P90) exposure to nutritional ketosis could impair learning and memory abilities. These data may suggest that the nutritional content of a KD is not sufficient to ensure proper neurodevelopment in young animals, while other studies argue that adequate composition of the diet (in terms of micronutrients and vitamins) is crucial for the health and development of young rodents fed with KD (26, 27, 75). We have previously demonstrated that modification of commonly used ketogenic chow allows for its application in developing rats, without causing detrimental side effects (27). Importantly, no adverse effects on neurodevelopment were observed in children using a KD to treat epilepsy (41). Hence, the observed underdevelopment and reduced cognitive abilities of young rodents fed with KD seem to be associated with inadequate composition of the diet or to be species-specific.

In animal models of TBI, the beneficial effect of the KD on cognition was demonstrated in four studies (45–48) while one study reported no effect (49). The KD-fed male adolescent (45–47) or young-adult rodents (47, 48) showed significantly improved recovery after injury and spatial memory in a variety of behavioral tests (MWM, NOR, or Y-maze) compared to the injured animals fed the control diet. Besides cognitive improvement, post-TBI KD administration resulted in better neurological outcomes including decreased degeneration of neurons in the dentate gyrus (45), attenuated neuroinflammation (46, 48), white matter damage, microgliosis (46), astrogliosis (45, 46), and oligodendrocyte loss (48), and improved sensorimotor functions (46, 48). Interestingly, Appelberg et al. (47) showed that KD introduced for 1 week immediately after TBI significantly improves cognitive recovery in adolescent rats but not in adult rats, suggesting that the effectiveness of ketones as an alternative fuel after TBI may be age-dependent. However, other authors demonstrate that applying an alternative KD formulation, with a fat-to-carbohydrate plus protein ratio of 2:1, containing MCT, docosahexaenoic acid, low glycemic index carbohydrates, fibers, and leucine, extends its neuroprotective potential in TBI to adult mice (48), again pointing out the importance of diet composition. One study reported that both pre-mild TBI and post-mild TBI exposure to the KD did not affect performance in novel context mismatch test in adolescent rats of both sexes. However, other parameters like balance and motor impairments, exploratory behavior, and telomere length were improved (49).

The observation that defective insulin signaling leading to decreased glucose metabolism may contribute to the progression of AD. It may be speculated that dietary interventions improving glucose and insulin metabolism might serve as a novel therapeutic approach to AD (76). Pre-clinical studies showed that the KD can mitigate some of the molecular and cellular changes associated with AD pathophysiology, resulting from enhancement in mitochondrial function, neuroprotection, reduction in neuroinflammatory response, and the expression of apoptotic mediators (21, 31). Moreover, the KD can help to eliminate brain amyloid-beta (Aβ) plaques by increasing the concentration of low density lipoprotein receptor-related protein 1 (LRP1), glycoprotein P (P-gp), and phosphatidylinositol binding clathrin assembly protein (PICALM) (77, 78). For instance, feeding with a KD decreased astroglial response to Aβ-plaques and lowered expression of the proinflammatory cytokines in the model of familial AD (79).

KD improved cognitive deficiency in female mice in a model of sleep deprivation-induced AD and a study with APP/PS1 mice, where KD was administered for 1 or 12 months (50, 51). While in female mice carrying the “London” APP mutation (APP/V717I) feeding with KD was not able to improve cognitive measures although it reduced Aβ40 and 42 levels by 25% (52). In other studies using genetic (APP/PS1, Tg4510) or pharmacological AD models (Aβ infusions) nutritional ketosis did not rescue memory deficits in a variety of experimental paradigms, varying in the time of KD exposure or diet composition (53, 54).

In addition to the aforementioned studies, there is further evidence indicating that nutritional ketosis can affect cognitive functions in animal models of neural disorders, stress, and obesity. KD administration improved cognition accompanied by histone modification in the model of neural disorders resulting from hypoxia injury (80), and Kmt2d+/βGeo mice (model of Kabuki syndrome) (81). A similar, positive effect of KD on cognition, related to peripheral metabolism (82) and biochemical changes in the hippocampus was shown in the rat model of chronic variable stress (83). We have previously shown that obesity-induced impairment in cognitive performance was ameliorated after weight loss achieved by either calorie restriction or KD. However, rats fed with a calorie-restricted KD performed better in MWM than those fed with a calorie-restricted standard diet (84). Finally, Fukushima et al. (55) concluded that the improvement in Y-maze performance may result from KD-induced increased hippocampal expression of the AMPA receptor subunit, GluR1 of naive adult rats. Also, the administration of KD with a ketogenic ratio of 6.6:0 has been shown to improve the Y-maze performance of naive adult rats in comparison to those fed with a standard diet. While no changes were observed with a ketogenic ratio of 3.0:0 (56). No effects of KD on cognition were reported in a few studies regarding synaptic functions (57–59), social behavior (60), evaluation of hippocampal involvement in spatial-cognitive behavior (61), or behavioral profiling (62) in wild type mice and rats.

Many studies suggest that a KD has a positive impact on cognition, especially in animal models of epilepsy, TBI, and aging. These studies generally report improvements, although sometimes there is no noticeable effect. The mitigation of cognitive impairment often goes hand in hand and may be secondary to other improvements in neurological and health outcomes such as reduced seizures in epilepsy models or reduced midlife mortality and improved health in old mice (64). However, in models of AD, the effects on cognition are usually moderate or even absent, despite reductions in amyloid deposition and other processes contributing to disease progression. In naive adult rodents, six articles report no effect (57–62), while two note cognition enhancement (55, 56). Four publications show that the application of a KD directly after weaning results in cognitive impairments later in life (40, 41, 71, 72).

### 3.2 Depressive-like behavior

The potential use of a KD in the treatment of depression is explored in the literature. Mechanisms through which a KD may potentially positively influence depression symptoms involve the modulation of the glutamate-glutamine cycle, gamma-aminobutyric acid (GABA) neurotransmission and, monoamine levels (85). Additionally, the diet provides nutrients such as ω-3 fatty acids, which may contribute to improvements in depression, and it may also influence the composition of the gut microbiota (86). It was proposed that KD through modulation of gut bacteria and its metabolites improves gut dysbiosis, decreases cytokine production, and lowers overall inflammation observed in depression (87). Another important finding underlying the therapeutic potential of nutritional ketosis comes from metabolic and behavioral analysis of Dravet mice fed with KD (44). KD reduced preference for saccharin in the sucrose preference test (SPT) in wild-type and Dravet mice, however, hippocampal levels of glutamate precursor ⍺-ketoglutarate and ⍺-D-glucose-1-phosphate correlated positively with saccharin preference in Dravet but not in wild-type mice (44). The SPT bases on natural rodents’ preference to selectively drink sweet solution when given a two-bottle free-choice regimen with access to both sucrose solution and water. A reduction in the sucrose preference ratio is indicative of anhedonia used for detection of depressive-like behavior in rodents (88).

The influence of nutritional ketosis on depressive-like behaviors and stress response was investigated in a few experimental studies (49, 57, 71, 89–96). Four studies have reported that nutritional ketosis may positively impact depressive-like behaviors (89–91, 93), as measured by the forced swim test (FST) and tail suspension test (TST), two classical behavior paradigms designed to measure depression levels, including changes observed in response to acute stress (97). The prolonged immobility in TST and FST are used for estimating depression-related behavior. Both of these models work similarly in assessing behavioral symptoms of feeling despair but not anhedonia (98).

One of the studies revealed that KD-fed rats exhibited less immobility duration in the FST when compared to those fed the standard diet (89). Additionally, the other study found that young adult CD-1 mice-offspring of mothers fed with a KD during pregnancy, exhibited reduced susceptibility to anxiety and depression (93). These observations were confirmed by Arqoub et al. (90) who observed that gestational exposure to KD reduced the expression of depressive-like behaviors in the FST. The latest study by Guan et al. (91), revealed that KD treatment decreased immobility duration in the TST and FST, and increased sucrose preference in the anhedonia-based SPT in repeated social defeat stress (R-SDS) and lipopolysaccharide (LPS) depression models. In the most recent paper by Gumus et al. (94), a combination of regular voluntary exercise with a KD decreased depressive-like behaviors in adult male mice, which was correlated with a decline of insulin and glucose or low/high-density lipoprotein (LDL/HDL) ratio and an increase of BHB levels. To the best of our knowledge, no studies are reporting the worsening of depressive-like behaviors in nutritional ketosis. No influence of KD feeding on depressive-like behaviors was reported in naive animals (57, 95), and the genetic model of Fragile X Syndrome (96).

The results from experimental studies suggest that nutritional ketosis may exhibit a beneficial influence on depressive-like behaviors. Considering the applied experimental paradigms – application of diet before acute stress or *in utero* - the effect may have a preventive character. Moreover, the differences observed in the experimental designs (e.g., the type of diet used, and time of administration), further emphasize the pressing need to investigate the underlying biological mechanisms of the anti-depression effects of the KD. Considering the positive impact of KD on depressive-like behavior, it might be postulated that nutritional ketosis might be used in depression treatment.

### 3.3 Anxiety-like behavior

The rationale behind the potential use of KD in anxiety disorders comes from its ability to counteract pathological changes in neurotransmission that are strongly linked to anxiety. These include GABA deficiency (99, 100) and increased neuronal excitability (15). The usefulness of the KD in anxiety disorders may also arise from its impact on gut microbiota, improvement of intestinal barrier function (101), its anti-inflammatory effects (102), and reduced production of reactive oxygen species (ROS) (103). The mechanisms substantiating the potential applicability of KDs in anxiety disorders were comprehensively discussed by Zhu et al. (15) and Wlodarczyk et al. (104) while the available clinical evidence was recently systematically reviewed by Dietch et al. (105).

Numerous behavioral tests have been developed to measure anxiety in rodents (106–108). In tests like open field, dark/light compartment tests, or elevated plus maze (EPM) the assessment of anxiety relies on the fact that laboratory rodents prefer closed and dark over open and light spaces (109). Other popular type of tests, used mostly for anxiolytic screening, are “conflict” tests like the Geller-Seifter or Vogel test, in which a hungry or thirsty animal is given an option to obtain, respectively, food or water by pressing a lever that can also elicit electric shock (110, 111). Due to the nature of anxiety tests separating anxiety, exploratory, activity, and learning responses is often not possible. Therefore, for the interpretation of results and understanding of their translational potential, it is crucial to recognize that a multitude of factors influences animal behavior in those tests. The overview of most common animal tests of anxiety alongside the consideration of conceptual issues regarding methodological details, interpretation of results, and intraspecies translation is comprehensively discussed in excellent reviews that focus on anxiety evaluation in preclinical settings (106–108).

Twenty articles evaluating the influence of nutritional ketosis on anxiety-related behaviors met the eligibility criteria for this review. Most data indicate that the KD does not influence anxiety-related behaviors (39, 55, 57, 60, 64, 69, 92, 95, 96, 112–115).

Among studies performed on rats, two reported positive effects of the KD on anxiety. Both young and aged naive rats fed with a KD showed resilience against the anxiogenic open arm in the EPM test (68). KD treatment showed protective properties by reducing anxiety levels in a model of TBI. Rats exposed to the KD post-injury showed reduced anxiety- and depressive-like behaviors acutely post-TBI. While pre-injury exposure to the KD resulted in even more pronounced improvement of outcomes like reduced balance and motor impairments (49). Interestingly, one study reported increased anxiety levels and decreased locomotor activity on a KD that were reversed by environmental enrichment (71). Other studies performed on rats report no effect of a KD on anxiety-related behaviors (39, 60, 95, 112, 113).

In naive mice, one study reported that a combination of a KD and regular voluntary exercise ameliorated anxiety and depression-like behaviors (Balb/c mice) (94). However, other studies performed on naive mice did not show changes in anxiety-related behaviors (55), also in the context of aging, in male and female mice, despite improvements in other neurocognitive functions (54, 69). Gestational exposure to a KD resulted in reduced susceptibility to anxiety and depression in adulthood, alongside many neuro-anatomical differences (93). Reduced anxiety under nutritional ketosis was also reported in a study performed on a model of ASD in BTBR mice (116). Moreover, a general improvement of autism symptoms after KD treatment was observed in numerous preclinical models of ASD (10, 116–119). Other research showed that a KD supplemented with ketone monoester reduced handling-induced convulsions and anxiety-like behaviors in early alcohol withdrawal (120). The tendency toward lower anxiety-like behaviors was reported in other studies performed on mice including the model of MPC1 deficiency in adult glutamatergic neurons (114) or Fragile X Syndrome (96), but none of the studies reported anxiogenic effects of nutritional ketosis. Interestingly, a study evaluating the effects of chronic or subchronic (7 days) administration of exogenous ketones alongside a standard diet reported a reduction of anxiety assessed with EPM test in all treatment conditions (121). Summing up, studies evaluating anxiety-related behaviors in nutritional ketosis report either no effect or reduced anxiety in the majority. The latter seems to be an indirect effect originating from the mitigation of pathophysiological changes specific to the examined disease model.

### 3.4 Social behavior

Given the beneficial effect of nutritional ketosis on epilepsy, mitochondrial function, carbohydrate metabolism, and inflammation, it has been proposed that treatment with a KD has the potential to reduce some of the ASD-associated symptoms, including impaired social interactions (122). The social behavior of laboratory rodents is most commonly evaluated with a 3-chamber test which allows for assessing sociability (time spent in the chamber with mouse vs. chamber with object) and preference for social novelty (time spent with unknown vs. known mouse). Other commonly used tests include: social transmission of food preference or analysis of the social activity in a home cage where behaviors like sniffing and following are analyzed, or in the case of juvenile rodents, also play responses like evasion or rotation (123, 124). The influence of the KD on social behavior has been tested in nine experimental studies (10, 57, 60, 90, 115–119). Studies conducted in rodent models of ASD, i.e., the BTBR model (116), the prenatal valproic acid (VPA) model (117), Shank^3+/ΔC^ mice (115), *Engrailed* 2 null mice (10, 118), and the maternal immune activation model of ASD (119) reported improvement of social deficits. An increase in social activity has also been reported in wild-type rats fed with a KD (60, 117) and in offsprings of dams fed a KD during gestation (90). Only one study reported that feeding with KD has not affected sociability in naive mice (57). Despite differences in the used models, age of the animals, time of the treatment, and employed behavioral tests, most of all these studies coherently show the increased social activity of rodents fed with the KD. This suggests that the mechanism by which the KD increases social activity is independent of the alterations underlying social impairment. Another line of evidence supporting this conclusion will be the observation that KD-induced reduction in social impairment in BTBR mice is not secondary to the well-known antiepileptic properties of this diet (116). It can be hypothesized that increased social activity results from other behavioral changes like increased arousal which translates to greater locomotor activity or reduced anxiety which leads to enhanced interest in the environment in general. However, neither reduced anxiety, increased locomotor activity nor changes in memory were reported in the studies mentioned above (10, 60). The effect is not persistent since the level of social activity is restored to control levels after the cessation of the diet (10, 60). In contrast, KD ameliorated autism-like social deficits observed in Shank^3+/ΔC^ mice, and this positive impact endured for up to 6 weeks after discontinuation of the diet (115). Most of the above-mentioned studies were conducted on male rodents, however, it has been also demonstrated that in *Engrailed* 2 null mice KD improved multiple measures of sociability in females, with limited effects in males (118). Although mechanisms underlying changes in social behavior in nutritional ketosis have not been deeply investigated, some insight was provided by Verpeut et al. (10) in the study performed on *Engrailed* 2 null mice, where immunohistochemical analysis demonstrated that groups exposed to the KD, regardless of genotype, showed increased neuronal activation in response to novel animal exposure. The KD-fed animals had more c-Fos positive cells in brain regions associated with social behaviors including the cingulate cortex, lateral septal nuclei, and anterior bed nucleus of the stria terminalis (10). This supports the idea that an increase in various aspects of sociability observed in the abovementioned studies arises from the impact of the KD on neuronal circuits controlling social behavior, independently of particular pathology underlying social impairment in used disease models.

### 3.5 Nutritional behavior

The KD is increasingly used for the treatment of obesity and as an add-on therapy in the management of type 2 diabetes (T2DM) (125). Meta-analyses comparing the effectivity of KDs to low-fat diets consistently show slightly greater weight loss, improved HDL-cholesterol, triacylglycerol (TAG), and other cardiometabolic markers but increased LDL-cholesterol (126–128). Although there is no consensus on the precise mechanism that determines the efficiency of weight loss under nutritional ketosis suppression of appetite is considered the play a leading role (17, 129, 130). Increased feeling of hunger is a common side effect of diet-induced weight loss that in the long term leads to reduced patient adherence compromising the results of the therapy and finally promoting weight regain (131, 132). Therefore understanding the mechanism of appetite suppression on a KD can significantly contribute to the improvement of weight loss therapies both in terms of lifestyle intervention as well as the development of new drugs. Studying the appetite-controlling neuroendocrine network on molecular and cellular levels is almost exclusively possible with the use of animal models. However, a question arises if animal models are suitable for studying appetite regulation under nutritional ketosis, i.e., if reduction of appetite occurs in laboratory rodents fed with the KD and how it can be measured, and finally what are the best experimental conditions to reflect the human situation. In the field of nutrition obesity on the behavior of rodents is most commonly evaluated by measuring food intake expressed in grams or in calories if different diets are compared. The most common methods for quantifying food consumption are manual weighing of the chow, but also automated chow counters, pellet dispensers, or video monitoring (133). The important factor compromising the accuracy of these methods is the fact that not the whole amount of chow leaving the food containers is consumed by animals. Especially in the case of high-fat diets (HFD) due to their consistency the chow crumbles (or is shredded by the animals) and falls into the bedding. This impacts the reliability of comparison between chows having various consistencies like standard chow and ketogenic chow. Another aspect important for methodological considerations is performing food deprivation studies or using food as a reward or reinforcement. Inappropriately chosen snack may lower the level of nutritional ketosis. Moreover, animals fed with different diets (standard and KD) may vary in their interaction with food, e.g., the motivation to obtain food, or may have different fasting tolerance. In the first couple of days after switching to a KD rodents usually reduce food/calorie intake (134–136). This reflects the need to acclimate to the type and consistency of new chow rather than a reduction of appetite since the calorie intake quickly goes back to baseline levels (134–136). After the habituation period, rodents fed with a KD usually consume less food expressed in grams but equal calories as the animals fed with standard chow. This trend was observed in wild-type animals fed with KD for up to 2 months (26, 55, 57, 83, 137–141) or longer (58, 142–145) as well as in different disease models like T2DM model (146), AD model (54), in the stress model (92), glaucoma model in both females and males (147), models of hepatic enzyme disturbances (148, 149), and in acute alcohol withdrawal symptoms (120). However, despite the equivalent caloric intake, feeding with KD often results in improved body mass (26, 83, 138, 140, 144) and metabolic health in long-term treatment (65). In some studies, rodents fed with KD consumed more calories than chow-fed controls, which was probably associated with lower energy assimilation because weight gain was not increased (44, 60, 95, 135, 150–158). One exception is a study in the model of Dravet syndrome where increased calorie intake was accompanied by increased body weight probably resulting from improvement of other disease symptoms (44). Decreased calorie intake, accompanied by decreased body weight during nutritional ketosis was reported in a couple of studies (53, 159–163). Taken together the majority of studies show that a KD, in comparison to standard chow, does not induce a spontaneous reduction in food intake. This suggests that the reduction in appetite observed in humans following a KD either does not occur in laboratory rodents, at least when compared to a standard chow, or is not detected through routine monitoring of food intake.

To answer the question of whether rodent models can be used to study mechanisms of appetite regulation under nutritional ketosis assessment of appetite could be performed in conditions where animals significantly overeat. This can be achieved by testing the changes in the appetite of diet induced obesity (DIO) animals after switching them to a standard or KD or by offering palatable snacks to animals fed with either KD or standard diet. It is a matter of ongoing discussion whether the disruption of homeostatic hunger regulation or the hedonic reward system governs overeating in rodents exposed to HFDs (164).

Therefore, experimental conditions need to be planned carefully with consideration of the underlying neuroendocrine status, choice of food/snacks, and test methods. Not only monitoring food intake but also applying behavioral tests like the food/risk competition test or food preference test would be informative (165). According to our best knowledge, such studies have not been performed so far. However, one study showed that animals previously fed with KD or HFD preferred to obtain morecalories from HFD, than animals fed with standard chow (55).

Laboratory rodents are widely used in research exploring mechanisms of KD action. However, the aspect of appetite regulation was not addressed in those studies so far and it remains an open question whether animal models are adequate research tools in this regard. Multiple factors including the differences in feeding patterns of humans and laboratory rodents or mechanisms that govern overeating need to be taken into account. Although study design seems to be challenging, the potential benefits of understanding KD-induced changes in the neuroendocrine network controlling appetite may have implications for the treatment of obesity that go beyond the use of the KD itself.

## 4 Summary

The majority of research performed in various disease models shows that the KD positively impacts cognition, especially in the models of epilepsy, TBI, and aging but also in naive animals (Supplementary Table S1). Almost an equal number of studies reports a reduction or no effect of the KD on depressive-related behaviors.

For anxiety-related behaviors, the majority of studies show no effect of the KD treatment. The adverse influence of the KD on cognition, anxiety-, and depressive-related behaviors was rarely reported (Table 1). Beneficial effects observed in behavioral measures seem to arise from the neuroprotective properties of the KD. Since the behavioral changes are accompanied by improvements in other outcomes specific to a disease model. In contrast, the increase in social activity seems to be unspecific and independent of the underlying cause of social impairment.

**Table 1:** The behavioral effects of ketogenic diet treatment.

		Behavioral outcome					
	Research area	Positive effect	*n*	No effect	*n*	Negative effect	*n*
Cognition	Naive animals	(55, 56)	2	(57–62)	6	–	–
	Epilepsy	(37–39, 36)	4	(42–44)	3	(40, 41)	2
	Traumatic brain injury	(45–48)	4	(49)	1	–	–
	Aging	(63–70)	8	–	–	–	–
	Alzheimer’s disease	(50, 51)	2	(52–54)	3	–	–
	Neurodevelopment	–	–	–	–	(40, 41, 71, 72)	4
	Other	(80–84)	5	–	–	–	–
Depression-related behavior	Naive animals	(71, 89)	2	(86, 95)	2	–	-
	Stress	(91)	1	(92)	1	–	–
	Prenatal exposure	(90, 93)	2	–	–	–	–
	Other	(49, 95)	2	(96)	1	–	–
Anxiety-related behavior	Naive animals	–	–	(55, 57, 60, 95, 112)	5	–	–
	Epilepsy	–	–	(39)	1	–	–
	Traumatic brain injury	(49)	1	–	-	–	–
	Aging	(68)	1	(64, 69, 113)	3	–	–
	Neurodevelopment	–	–	–	–	(71)	1
	Stress	–	–	(92)	1	–	–
	Prenatal exposure	(93)	1	-	-	–	–
	Autism spectrum disorders	(116)	1	(115)	1	–	–
	Other	(94, 120)	2	(96, 114)	2	–	–
Social behavior	Naive animals	(60, 90)	2	(57)	1	–	–
	Autism spectrum disorders	(10, 115–119)	6	–	–	–	–

Despite the growing use of the KD in the treatment of obesity, accompanied by scientifically proven efficiency and appetite-reducing properties of the KD, the aspect of nutritional behavior of KD-fed animals has not been addressed so far. It remains an open question whether animal models are adequate research tools to study appetite regulation under nutritional ketosis.

Using animal models to evaluate the influence of the KD on behavioral measures requires paying special attention to the composition of the diet. While the proper macronutrient ratio is reflected by elevated blood BHB levels, an adequate micronutrient supply must be provided to avoid malnutrition that will affect behavioral measures (26, 27, 75). A couple of studies point out that additional supplementation of the KD with, e.g., MCT, ketogenic amino acids, or exogenous ketones may impact not only physiological but also behavioral effects of the diet in laboratory animals (48, 166) but also in humans (167). Although currently, many scientists acknowledge the significance of the KD’s composition in determining its properties, most studies primarily investigate its effects in comparison to standard rodent chow. The differences between KDs of various compositions are rarely tested. Therefore, drawing direct conclusions about the role of diet composition or the presence of a particular ingredient that would be backed by strong scientific evidence is usually not possible.

## Author contributions

KG: Investigation, Visualization, Writing – original draft. MG: Investigation, Writing – original draft. MP: Investigation, Writing – original draft. NP: Investigation, Writing – original draft. JJB: Conceptualization, Supervision, Writing – review & editing. MN-Ch: Investigation, Methodology, Project administration, Resources, Supervision, Writing – original draft, Writing – review & editing. DL: Conceptualization, Methodology, Project administration, Supervision, Writing – original draft, Writing – review & editing.

## Figures and Tables

**Table:** 

AD	Alzheimer’s disease
ASD	Autism spectrum disorders
Aβ	Amyloid-beta
BHB	Beta-hydroxybutyrate
DIO	Diet-induced obesity
EPM	Elevated plus maze
FST	Forced swim test
GABA	Gamma-aminobutyric acid
HDL	High-density lipoprotein
HFD	High fat diet
KD	Ketogenic diet
LDL	Low-density lipoprotein
LPS	Lipopolysaccharide
LRP1	Low density lipoprotein receptor-related protein 1
MCT	Medium chain triglycerides
MWM	Morris water maze
NOR	Novel object recognition
P-gp	Glycoprotein P
PICALM	Phosphatidylinositol binding clathrin assembly protein
PTZ	Pentylenetetrazol
R-SDS	Repeated social defeat stress
ROS	Reactive oxygen species
SPT	Sucrose preference test
T2DM	Type 2 diabetes
TAG	Triacylglycerol
TBI	Traumatic brain injury
TST	Tail suspension test
VPA	Valproic acid
