# Supplementary material for: Ketogenic diet and behavior: insights from experimental studies
Source: Front Nutr. 2024 Feb 8;11:1322509. doi: 10.3389/fnut.2024.1322509 (PMC10881757; doi:10.3389/fnut.2024.1322509)
Supplement: Supplementary file 1 [file Table_1.docx]

Supplementary Material

# Effects of ketogenic diet on cognitive parameters

| **Effects of KD on cognitive parameter** | **Studied parameter** | **Behavioral test paradigm** | **References** |
| --- | --- | --- | --- |
| positive | hippocampal memory | Novel Object Recognition | 36 |
|  | memory | Circular Barnes maze | 37 |
|  | learning and memory | Morris Water Maze | 38 |
|  | spatial memory | Morris Water Maze | 39 |
|  | spatial and object memory | Novel Object Recognition |  |
|  |  | Novel placement recognition |  |
|  | spatial and visual memory | Y-maze | 45 |
|  |  | Novel Object Recognition |  |
|  | spatial working memory | Y-maze | 46 |
|  | cognitive performance in adolescents | Morris Water Maze | 47 |
|  | spatial memory | Morris Water Maze | 48 |
|  | spatial memory | Morris Water Maze | 50 |
|  | spatial learning and memory | Morris Water Maze | 51 |
|  |  | Y-maze |  |
|  | spatial learning | Y-maze | 55 |
|  | spatial working memory | Y-maze | 56 |
|  | spatial working memory | Y-maze | 63 |
|  | spatial learning memory | Barnes maze |  |
|  | memory | Novel Object Recognition | 64 |
|  |  | Place avoidance |  |
|  | memory | Novel Object Recognition | 65 |
|  | cognitive performance | T-maze | 66 |
|  |  | Object recognition |  |
|  | cognitive performance | Biconditional association task | 67 |
|  | working memory | Random arm biconditional association task | 68 |
|  |  | Dual working memory/biconditional association task |  |
|  | spatial learning | Barnes maze | 69 |
|  | recognition memory | Two-object novel object recognition | 70 |
|  | spatial learning and memory | Morris Water Maze | 80 |
|  | hippocampal memory | Morris Water Maze | 81 |
|  | hippocampal-dependent cognition | Feature negative discrimination | 82 |
|  | spatial learning and memory | Morris Water Maze | 83 |
|  | short-term memory | Novel Object Recognition |  |
|  | cognitive performance | Morris Water Maze | 84 |
| no effect | acquisition memory | Morris Water Maze | 39 |
|  | associative learning | Morris Water Maze | 42 |
|  | visuospatial learning and memory | Morris Water Maze | 43 |
|  | cognitive performance | Novel Object Recognition | 44 |
|  | cognitive performance in adults | Morris Water Maze | 47 |
|  | short-term working memory | Novel Context Mismatch task | 49 |
|  | non-spatial memory | Novel Object Recognition | 52 |
|  | spatial and short-term memory | Morris Water Maze | 53 |
|  |  | Y-maze |  |
|  |  | Passive avoidance |  |
|  | spatial and contextual memory | Radial Arm Water Maze | 54 |
|  |  | Fear conditioning |  |
|  | spatial learning | Morris Water Maze | 55 |
|  | spatial memory consolidation | Morris Water Maze | 57 |
|  | spatial recognition memory | Y-maze |  |
|  | spatial learning and memory | Y-maze | 58 |
|  | contextual fear and auditory cue | Fear conditioning | 59 |
|  | working and recognition memory | Novel Object Recognition | 60 |
|  | spatial cognitive behavior | Morris Water Maze | 61 |
|  | learning and memory | Hebb Williams Maze | 62 |
|  |  | Passive avoidance |  |
|  | object recognition memory | Novel Object Recognition | 63 |
|  | short-term working memory | Y-maze | 69 |
|  | recognition memory | Novel Object Recognition |  |
|  | incidental learning and short-term memory | Novel Object Recognition | 72 |
|  | associative learning | Morris Water Maze | 81 |
|  | hippocampal-independent cognition | Simple Pavlovian discrimination | 82 |
|  | learning and memory | Passive avoidance | 83 |
| negative | visuospatial learning and memory | Morris Water Maze | 40 |
|  | spatial learning and memory | Morris Water Maze | 41 |
|  | learning | Morris Water Maze | 71 |
|  | spatial learning and reference memory | Morris Water Maze | 72 |
| KD - ketogenic diet | | | |
